# Cortical and ventricular brain retention of ^11^C‐PIB assessed by dynamic PET/CT and correlation with the Aβ1‐42/Aβ1‐40 ratio of amyloid peptides in the cerebral spinal fluid. Preliminary analysis in a healthy control volunteer population

**DOI:** 10.1002/alz.089223

**Published:** 2025-01-03

**Authors:** Julio F Jiménez‐Bonilla, Francisco J Gómez de la Fuente, Sara López‐García, María De Arcocha‐Torres, Virginia J Mendi‐Barcina, Carmen J Lage, Francisco J Martínez‐Dubarbie, Remedios J Quirce

**Affiliations:** ^1^ Universitary Hospital M Valdecilla, Santander, Cantabria Spain; ^2^ Hospital Universitario Marqués de Valdecilla – IDIVAL – University of Cantabria, Santander, Cantabria Spain

## Abstract

**Background:**

Preclinical Alzheimer’s disease may be linked to impaired cerebral amyloid clearance. We aim to obtain dynamic parameters of cortical and ventricular clearance of ^11^C‐PIB and compare them with CSF amyloid values in a population of healthy volunteers.

**Method:**

We evaluated 8 healthy volunteers (4 men and mean age: 64.2 years) without neurological or cognitive alterations by a dynamic PET/CT for 45' starting 40 min after administration of 370 MBq of ^11^C‐PIB. Cortical VOIs of 1 cm3 were drawn in the cerebellar, frontal, temporal, occipital, parietal, and posterior periventricular regions and in the posterior lateral ventricle obtaining SUVmean values at 45', 60' and 75'. For each area, clearance velocity slopes were calculated in the 40‐60' and 60‐75' temporal period and SUVr values at 45, 60 and 75' considering cerebellum cortex as reference region. Its correlation with the values of the Aβ1‐42/Aβ1‐40 ratio assessed in the cerebral spinal fluid (CSF) was analyzed.

**Result:**

Four of the eight controls showed a pathological Aβ1‐42/Aβ1‐40 ratio. The Aβ1‐42/Aβ1‐40 ratio correlated with SUVr values in all of the studied regions in the total group (n = 8). The best correlations were obtained at 40' in parietal cortex (r = ‐0.821), temporal cortex (r = ‐0.816), posterior periventricular region (r = ‐0.683) and frontal cortex (r = ‐0.617). The posterior ventricular region only showed correlation at 75' (r = ‐0.523). Significant correlations were observed with the slope of the first section (45‐60') in frontal (r = 0.796), occipital (r = 0.793), parietal (r = 0.556), temporal (r = 0.426), and especially in the periventricular region. (r = 0.964). However, when these parameters are compared between the subgroup of subjects with pathological Aβ1‐42/Aβ1‐40 ratio (n = 4) and with normal ratio (n = 4), these correlations worsen in all regions in the pathological group and improves in the normal group, especially in the temporal region (‐0.149 vs ‐0.924) and in periventricular (0.158 vs 0.844) at 60' and 75' respectively.

**Conclusion:**

In our control healthy population, there is a correlation between the dynamic parameters of cerebral clearance of ^11C^‐PIB and the CSF amyloid value, with differences between subjects with a pathological and normal CSF amyloid values. Larger studies are necessary to determine the value of these findings in the preclinical Alzheimer’s disease.

